# Immunogenicity and Efficacy of A/H1N1pdm Vaccine Among Subjects With Severe Motor and Intellectual Disability in the 2010/11 Influenza Season

**DOI:** 10.2188/jea.JE20150036

**Published:** 2016-06-05

**Authors:** Megumi Hara, Tomoyuki Hanaoka, Kazuhiro Maeda, Tetsuo Kase, Satoko Ohfuji, Wakaba Fukushima, Yoshio Hirota

**Affiliations:** 1 Department of Preventive Medicine, Faculty of Medicine, Saga University, Saga, Japan; 2 Bihoro Ryoiku Hospital, Abashiri, Hokkaido, Japan; 3 Department of Hygiene and Preventive Medicine, Showa University, School of Medicine, Tokyo, Japan; 4 Hokkaido University Center for Environmental and Health Sciences, Sapporo, Japan; 5 The Research Foundation for Microbial Diseases of Osaka University, Kannonji, Kagawa, Japan; 6 Osaka Prefectural Institute of Public Health, Osaka, Japan; 7 Department of Public Health, Faculty of Medicine, Osaka City University, Osaka, Japan; 8 Clinical Epidemiology Research Center, Medical Co. LTA, Fukuoka, Japan; 9 College of Healthcare Management, Fukuoka, Japan

**Keywords:** influenza vaccine, immunogenicity, antibody efficacy, vaccine efficacy, disabled person

## Abstract

**Background:**

While the immunogenicity and effectiveness of seasonal influenza vaccines among subjects with severe motor and intellectual disability (SMID) are known to be diminished, the efficacy of the A/H1N1pdm vaccine has not been evaluated.

**Methods:**

We prospectively evaluated 103 subjects with SMID (mean age, 41.7 years) who received trivalent inactivated influenza vaccine during the 2010/11 influenza season. The hemagglutination inhibition (HI) antibody titer was measured in serum samples collected pre-vaccination (S0), post-vaccination (S1), and end-of-season (S2) to evaluate subjects’ immunogenicity capacity. Vaccine efficacy was assessed based on antibody efficacy and achievement proportion.

**Results:**

The proportions of seroprotection and seroconversion, and the geometric mean titer (GMT) ratio (GMT at S1/GMT at S0) for A/H1N1pdm were 46.0%, 16.0%, and 1.8, respectively—values which did not meet the European Medicines Evaluation Agency criteria. The achievement proportion was 26%. During follow-up, 11 of 43 subjects with acute respiratory illness were diagnosed with type A influenza according to a rapid influenza diagnostic test (RIDT), and A/H1N1pdm strains were isolated from the throat swabs of 5 of those 11 subjects. When either or both RIDT-diagnosed influenza or serologically diagnosed influenza (HI titer at S2/HI titer at S1 ≥2) were defined as probable influenza, subjects with A/H1N1pdm seroprotection were found to have a lower incidence of probable influenza (odds ratio, 0.31; antibody efficacy, 69%; vaccine efficacy, 18%).

**Conclusions:**

In the present seasonal assessment, antibody efficacy was moderate against A/H1N1pdm among SMID subjects, but vaccine efficacy was low due to the reduced immunogenicity of SMID subjects.

## INTRODUCTION

Severe motor and intellectual disability (SMID) is defined as being bedridden or only able to sit, crawl, or walk with support and having a relatively low intelligence quotient (<35).^1^ Further, such individuals are generally debilitated and also immunocompromised,^2^ with a lower immunogenicity to seasonal influenza vaccines than healthy individuals and no booster effect; moreover, age has a greater influence on immunogenicity than on symptom severity in this population.^2^^,^^3^ We previously reported that, in the 2009 A/H1N1pdm influenza pandemic, a single dose of A/H1N1pdm monovalent vaccine did not induce sufficient immunity in individuals with SMID, and a second dose was likely to be ineffective as well, given the diminished immunogenicity capacity of this population.^4^ Because pre-vaccination levels of hemagglutination inhibition (HI) antibodies against the vaccine strain significantly influence immunogenicity,^5^ studies to determine whether or not immunogenicity improved in subjects with SMID in the following season are required.

Similar to immunogenicity, vaccine efficacy is also suspected to be diminished among subjects with SMID. However, vaccine efficacy is based on the percentage reduction in incidence of influenza in vaccinated subjects compared to that in unvaccinated subjects.^6^^,^^7^ Given that some 40%–60% of SMID subjects reside in chronic-care facilities in Japan^1^ and most are vaccinated, evaluating vaccine efficacy is difficult.

In such situations, an index of “antibody efficacy”^8^ is used, which was described by Longini et al in 1988 and reflects the percentage reduction in influenza incidence among subjects with a protective post-vaccination HI titer compared with that among subjects without this HI titer. Antibody efficacy has two important advantages over vaccine efficacy: it can estimate vaccine efficacy in any target population, even a population with 100% vaccination coverage, and it can do so blindly. Because pre-vaccination, post-vaccination, and end-of-season HI titers are analyzed simultaneously at the end of follow-up, the outcomes are evaluated without knowledge of the subjects’ HI titer status. An accurate determination of the incidence of strain-specific influenza is crucial to calculating antibody efficacy. However, only a few studies have used antibody efficacy to assess vaccine efficacy^9^^–^^11^ because of the difficulties involved in virological confirmation.

Here, to estimate vaccine efficacy of nonadjuvanted trivalent inactivated influenza vaccine (IIV3) including the A/H1N1pdm strain in subjects with SMID, we conducted a prospective observational study evaluating the immunogenicity and antibody efficacy of this vaccine during the 2010/11 influenza season.

## METHODS

### Study subjects and study season

Our study was conducted in Japan during the 2010/2011 influenza season. According to reports from the Hokkaido Infectious Disease Surveillance Center, as recorded by the National Epidemiological Surveillance of Infectious Disease, an influenza epidemic occurred between January 10 and May 28, 2011 in the Abashiri area, where the research facility was located. Circulating strains were antigenically well matched to A/H1N1pdm.^12^

Study subjects were 103 individuals with SMID who mainly suffered from cerebral palsy, epilepsy, and cognitive disorders and resided in a long-term care facility in Hokkaido Prefecture, located in northern Japan. They had received 2 doses of nonadjuvanted split-virus A/H1N1pdm vaccine containing at least 15 µg hemagglutinin antigen to A/California/7/2009 (A/H1N1)v-like strain from the 2009 pandemic (lot no. HP01A in 2009; Research Foundation for Microbial Disease of Osaka University, Osaka, Japan). None of the subjects had been infected with the influenza strain from the 2009/10 season.^4^

### Standard protocol approval, registration, and patient consent

The study subjects’ guardians provided written informed consent for their participation in our study. The baseline characteristics of subjects, including age, sex, and chronic medical conditions, were collected from medical records. None of the subjects had a history of allergy to eggs or anaphylaxis to vaccine components. The study protocol was approved by the Institutional Review Board of the Saga University Faculty of Medicine (H21-54) and was conducted in accordance with the Ethical Guidelines for Epidemiological Research of the Ministry of Education, Culture, Sports, Science and Technology and Ministry of Health, Labour and Welfare of Japan. The study was registered in the UMIN Clinical Trials Registry (UMIN000015037).

### Vaccination and serum specimen collection

All subjects received a single dose of 0.5 mL IIV3 (lot no. HA110A; Research Foundation for Microbial Disease of Osaka University) subcutaneously into their arm on November 1, 2010. The vaccine contained at least 15 µg each of hemagglutinin antigen to A/California/7/2009 (H1N1)pdm, A/Victoria/210/2009 (H3N2), and B/Brisbane/60/208. After vaccination, healthcare workers at the facility carefully observed vaccinated subjects for anaphylactic shock for at least 30 minutes and adverse reactions for 48 hours following vaccination, including either local (erythema, swelling, induration, itching, and pain) or general reactions (fever, fatigue, myalgia or arthralgia, headache, and rash).

Serum samples were collected before vaccination (S0), three weeks after vaccination (S1), and six months after vaccination (S2). All serum samples were stored at −40°C until assayed.

### Measurement and evaluation for immunogenicity

The serum antibody titer against the vaccine strain was measured routinely using the HI assay with chicken erythrocytes.^13^^,^^14^ Serum samples were treated with receptor-destroying enzyme (*Vibrio cholera* filtrate; Denka Seiken, Tokyo, Japan) to inactivate nonspecific inhibitors. All samples were assayed simultaneously at the laboratory of the Research Foundation for Microbial Disease of Osaka University.

The geometric mean titer (GMT) of HI, seroprotection proportion (proportion of subjects with HI titer ≥1:40 at S1), and seroconversion proportion (proportion of subjects with HI titer <1:10 at S0 and ≥1:40 S1, or ≥1:10 at S0 with a 4-fold increase in titer at S1 compared with that at S0) were calculated. If HI titers were below or above the detection limits (<1:10 or >1:5120), they were set as 1:5 or 1:5120, respectively. The GMTs at S1 were compared with those at S0, and the GMT ratio was calculated. The achievement proportion was calculated as the proportion of subjects with an HI titer <1:40 at S0 and ≥1:40 at S1.

Immunogenicity was evaluated according to the European Medicines Evaluation Agency (EMEA) criteria for evaluating HI antibody responses to seasonal vaccine.^15^ The cut-off values for vaccine immunogenicity in adults aged 18–60 years were a seroprotection proportion >70%, seroconversion proportion >40%, or mean geometric increase >2.5.

### Follow-up and definition of outcome

After vaccination, all subjects were followed from the November 1, 2010 to May 31, 2011. Healthcare workers measured subjects’ body temperature every morning and afternoon and prospectively recorded respiratory symptoms (cough, sore throat, and nasal congestion) and other general symptoms (fever, muscle pain, and general fatigue). When subjects had a fever (body temperature ≥37.8°C), throat swabs were collected and tested using a rapid influenza diagnosis test (RIDT; Capilia FluA, B; Becton-Dickinson Japan, Tokyo, Japan), based on an immunochromatographic method. If the test was positive for infection, throat swabs collected from the patients were stored at −40°C. To confirm the existence and strain of the influenza virus in potentially infected patients, we cultured the circulating influenza virus using standard methods at the Osaka Prefectural Institute of Public Health laboratory.

We established six outcomes for vaccine effectiveness: acute respiratory illness (ARI), defined as sudden-onset fever (body temperature ≥37.8°C)^16^; influenza-like illness (ILI), defined as ARI within the influenza epidemic period when RIDT-diagnosed influenza cases were observed (from January 17 to February 6); RIDT-diagnosed influenza; serologically diagnosed influenza 1 (HI titer at S2/HI titer at S1 ≥4); serologically diagnosed influenza 2 (HI titer at S2/HI titer at S1 ≥2); and probable influenza, defined as RIDT-diagnosed influenza and an RIDT-negative result with serologically diagnosed influenza 2.

### Statistical analysis

All statistical analyses were performed using SAS 9.3 for Windows (SAS Institute, Cary, NC, USA). Regarding immunogenicity, the 95% confidence intervals (CIs) of seroprotection and seroconversion proportions were calculated using the exact binomial distribution for proportions. Wilcoxon’s rank-sum test was used to compare GMT and GMT ratios between groups, while Wilcoxon’s signed-rank test was used to determine the significance of the increase in HI antibody titers post-vaccination in each group. The baseline characteristics and seroprotection proportion were compared using the chi-square test or Fisher’s exact test.

To clarify confounding factors for antibody efficacy, we initially examined factors associated with both seroprotection and outcomes. Odds ratios (OR) and 95% CIs of subjects exhibiting post-vaccination seroprotection for each outcome were then calculated by multiple logistic regression, with adjustment for possible confounding factors. Antibody efficacy was calculated as follows: [1 − adjusted OR] × 100%. The product of antibody efficacy and achievement proportion is theoretically equivalent to vaccine efficacy.^9^

## RESULTS

A total of 103 subjects with SMID (56 men and 47 women; mean age, 41.7 [standard deviation, 10.4] years) were institutionalized with one or more of the following: cerebral palsy (*n* = 45), epilepsy (*n* = 29), intelligence impairment (*n* = 8), post-meningitis (*n* = 6), and other reasons (*n* = 25). Subjects had no influenza infection within one month or three weeks after vaccination. No serious adverse events occurred during the study period. While several mild adverse reactions, such as local redness and swelling, were reported, these were transient.

Subjects’ immunogenicity to A/H1N1pdm did not meet the EMEA criteria (Table 1). The seroprotection proportion was 46%, the seroconversion proportion 16%, and the GMT ratio 1.8, with an achievement proportion of 26%. Age was not associated with immunogenicity. The GMT ratio was lower in male subjects than in female subjects, and the seroprotection proportion was significantly lower in those with asthma than in those without (*P* = 0.02). Subjects with a higher pre-vaccination HI titer had a lower GMT ratio and higher proportion of seroprotection than those with relatively low pre-vaccination titers. Subjects with a pre-vaccination HI titer of 1:10–20 had a higher seroconversion proportion and achievement proportion than those with an HI titer <1:10.

**Table 1.** Immunogenicity against A/H1N1pdm among subjects

	*n*	GMT		GMT ratio		Seroprotection	Seroconversion	Achievement	
				
		Pre (S0)	Post (S1)	S1/S0		*n* (%, 95% CI)	*n* (%, 95% CI)	*n*^a^	*n* (%, 95% CI)
Total	103	16	29	1.8	(*P* < 0.0001)	47 (46, 36–55)	16 (16, 9–23)	73	19 (26, 16–36)

Sex									
Male	56	15	24	1.6	(*P* < 0.0001)	21 (38, 25–50)	6 (11, 3–19)	41	8 (20, 9–32)
Female	47	17	36	2.2	(*P* < 0.0001)	26 (55, 41–70)	10 (21, 10–33)	32	11 (34, 19–53)
		(*P* = 0.77)	(*P* = 0.07)	(*P* = 0.04)		(*P* = 0.08)	(*P* = 0.18)		(*P* = 0.18)

Age, years									
<30	23	19	41	2.1	(*P* < 0.0001)	13 (57, 36–77)	4 (17, 2–33)	13	3 (23, 5–54)
30–39	21	18	31	1.8	(*P* < 0.0001)	11 (52, 31–74)	6 (29, 9–48)	17	8 (47, 23–72)
40–49	23	13	25	1.9	(*P* < 0.0001)	10 (43, 23–64)	2 (9, 0–20)	16	3 (19, 4–46)
≥50	36	15	24	1.6	(*P* < 0.0001)	13 (36, 20–52)	4 (11, 1–21)	27	5 (19, 6–38)
		(*P* = 0.63)	(*P* = 0.25)	(*P* = 0.50)		(*P* = 0.42)	(*P* = 0.25)		(*P* = 0.25)

Asthma									
Without	93	17	30	1.8	(*P* < 0.0001)	46 (49, 39–60)	16 (17, 10–25)	64	19 (30, 19–42)
With	10	9	17	2.0	(*P* = 0.0002)	1 (10, 0–29)	0 (0, 0)	9	0 (0, 0–34)
		(*P* = 0.04)	(*P* = 0.10)	(*P* = 0.29)		(*P* = 0.02)	(*P* = 0.35)		(*P* = 0.05)

Pre-vaccination HI titer against A/H1N1									
<1:10	33	5	12	2.4	(*P* < 0.0001)	2 (6, 0–14)	2 (6, 0–14)	33	2 (6, 1–20)
1:10–1:20	40	14	26	1.9	(*P* < 0.0001)	17 (43, 27–58)	11 (28, 14–41)	40	17 (43, 27–59)
≥1:40	30	65	84	1.3	(*P* = 0.0625)	28 (93, 84–100)	3 (10, 0–20)		(*P* < 0.01)
		(*P* < 0.01)	(*P* < 0.01)	(*P* < 0.01)		(*P* < 0.01)	(*P* = 0.61)		

SMIDs, severe motor and intellectual disabilities; GMT, geometric mean titer; GMTR, GMT ratio; CI, confidence interval.

Comparisons with pre-vaccination data were made using Wilcoxon’s signed-rank test, and comparisons between categories were made using Wilcoxon’s rank-sum test, Wilcoxon’s signed-rank test, Chi-square test, or Fisher’s exact test.

^a^Number of subjects with a pre-vaccination HI antibody titer <1:40.

Figure shows the numbers of ARI cases and RIDT-diagnosed influenza cases in this study population during the observation period. Among 43 cases of ARI, 11 were diagnosed as type A influenza by RIDT, and of these 11, 5 were virologically confirmed to have A/H1N1pdm influenza.

**Figure.**
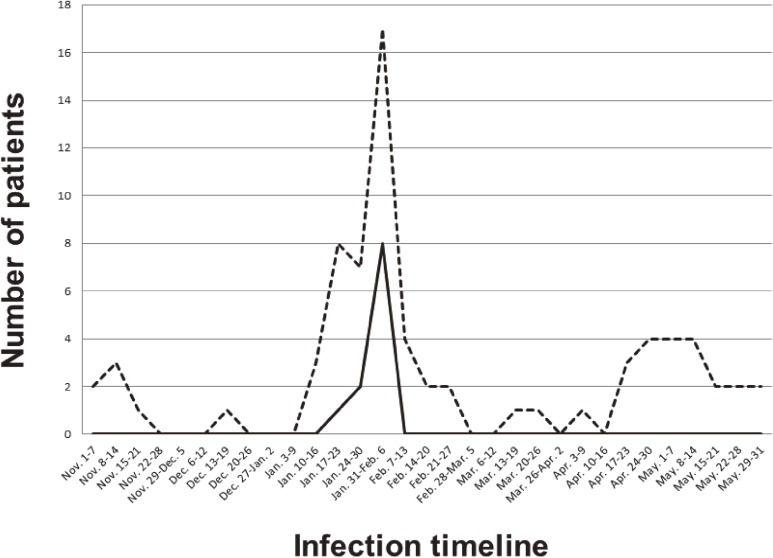
Numbers of ARI (dotted line) and RIDT-diagnosed influenza (solid line) cases during the observation period. ARI, acute respiratory illness; RIDT, rapid influenza diagnosis test.

Given that asthma was associated with a reduced seroprotection proportion against H1N1pdm (OR 0.11; 95% CI, 0.01–0.93) and with every outcome (eTable 1), asthma was considered a confounding factor for antibody efficacy. The crude and asthma-adjusted ORs of seroprotection, antibody efficacy, and vaccine efficacy against A/H1N1pdm for the six outcomes are summarized in Table 2. Asthma-adjusted ORs were decreased when more specific outcomes were used, with values of 0.82 (95% CI, 0.36–1.88) for ARI, 0.58 (95% CI, 0.20–1.63) for ILI, and 0.52 (95% CI, 0.12–2.24) for RIDT-diagnosed influenza. The asthma-adjusted OR of serologically diagnosed influenza 1 was lower than that of serologically diagnosed influenza 2. The asthma-adjusted OR of probable influenza was 0.31 (95% CI, 0.08–1.22). Given the above findings, the antibody efficacy and vaccine efficacy were determined to be 69.1% and 18.0%, respectively.

**Table 2.** Effectiveness of influenza vaccine for influenza-related outcomes evaluated based on asthma-adjusted ORs, antibody efficacy, and vaccine efficacy

Outcomes	Post-vaccination		Crude OR	95% CI	Asthma-adjusted OR	95% CI	Antibody Efficacy (%)^a^	95% CI	Vaccine Efficacy (%)^b^	95% CI

	HI < 40	HI ≥ 40								
	(*n* = 56)	(*n* = 47)								
ARI (fever ≥37.8°C)	26	17	0.65	(0.30 to 1.45)	0.82	(0.36 to 1.88)	18.0	(−88 to 64)	4.7	(−22.9 to 16.6)
ILI (ARI within influenza endemic period)	16	7	0.44	(0.16 to 1.18)	0.58	(0.20 to 1.63)	42.4	(−63 to 80)	11.0	(−16.4 to 20.8)
RIDT-diagnosed influenza	8	3	0.41	(0.10 to 1.64)	0.52	(0.12 to 2.24)	47.6	(−124 to 88)	12.4	(−32.2 to 22.9)
Serologically diagnosed influenza 1^c^	12	2	0.16	(0.03 to 0.77)	0.20	(0.04 to 0.99)	79.8	(1 to 96)	20.7	(0.3 to 25.0)
Serologically diagnosed influenza 2^d^	14	4	0.28	(0.09 to 0.92)	0.33	(0.10 to 1.11)	67.4	(−11 to 90)	17.5	(−2.9 to 23.4)
Probable influenza^e^	12	3	0.25	(0.07 to 0.95)	0.31	(0.08 to 1.22)	69.1	(−22 to 92)	18.0	(−5.7 to 23.9)

ARI, acute respiratory illness; CI, confidence interval; HI, hemagglutination inhibition; ILI, influenza-like illness; OR, odds ratio; RIDT, rapid influenza diagnosis test; S1, post-vaccination; S2, end of the season.

^a^[1 − asthma-adjusted OR] × 100%.

^b^Antibody efficacy × achievement proportion (0.26).

^c^HI titer at S2/HI titer at S1 ≥4.

^d^HI titer at S2/HI titer at S1 ≥2.

^e^RIDT-diagnosed influenza and an RIDT-negative result with serologically diagnosed influenza 2.

## DISCUSSION

The immunogenicity of the A/H1N1pdm vaccine strain among subjects with SMID was deemed insufficient based on international standards, even after vaccination in the second season. Priming as a result of prior exposure to a related influenza strain through infection or immunization is well known to promptly induce a potent antibody response to immunization.^5^ Preexisting memory T and B cells are involved in rapid and strong responses to a second vaccination, and memory T cells are crucial for controlling humoral and cellular immune responses.^17^ The SMID subjects in the present study were considered to have diminished immunogenicity, given their decreased cellular immune responses and lack of a history of A/H1N1pdm influenza infection in the 2009/10 influenza season. A high pre-vaccination HI titer generally contributes to higher seroprotection, and our present findings confirmed that this association held true even among subjects with SMID. In contrast, a high pre-vaccination HI titer is generally associated with lower seroconversion proportions. However, in the present study, seroconversion proportions were the lowest in subjects with no detectable antibody levels at pre-vaccination despite receiving an A/H1N1pdm vaccine the previous influenza season. Taken together, these results indicate that non-responders to A/H1N1pdm vaccination have diminished cellular function, such as impaired immune memory or reduced immune response.

In the present study, immunogenicity was significantly diminished among subjects with a history of asthma, although details regarding their asthma treatment were unknown. Inhaled steroid hormones are usually used as preventive medication for asthma attacks, and injection or oral steroids are used for controlling attacks. Given that steroids are considered to affect T-cell immunity, steroid treatment may reduce vaccine immunogenicity.^18^ Several studies have investigated the immunogenicity of inactivated influenza vaccine among asthma patients. Hanania et al reported that immune response to the A antigens of IIV3 in asthma patients was not adversely affected by inhaled corticosteroids,^19^ and Bae et al reported that IIV3 induced a protective immune response in children with recurrent wheezing requiring frequent steroid treatment.^20^ While these previous findings conflict with our own, Bae et al’s study was conducted among children, and Hanania et al’s study was conducted among children and adults. In contrast, Busse et al reported that patients aged more than 60 years with severe asthma had lower immunogenicity than younger patients with severe asthma, and their immunogenicity did not meet the EMEA criteria.^21^ Further, SMIDs have been reported to be associated with rapid physical aging and degeneration.^2^ Our present findings concur with those of Busse et al, as asthma was related to lower immunogenicity. In addition, asthma condition was associated with outcomes, as SMID patients with asthma had significantly higher ORs for ARI, ILI, serologically diagnosed influenza 1, and probable influenza than those without asthma (eTable 1). We therefore conducted multivariate analysis to control for its confounding effects.^22^

To our knowledge, ours is the first study to evaluate the effectiveness of IIV3 that includes A/H1N1pdm among subjects with SMIDs. Although no statistical significance was observed for most outcomes due to limited power, the ORs of seroprotection were <1 for all outcomes, suggesting relatively low influenza incidence in the population. Because misclassification of influenza generally reduces vaccine effectiveness, specific definitions for outcomes must be used to increase accuracy of influenza diagnosis.^23^ When we limited ARIs to ILI or RIDT-diagnosed influenza, adjusted ORs were decreased. Although the RIDT has high specificity and moderate sensitivity, its accuracy depends on virus count, resulting in the potential for false negatives. For instance, time since fever onset and sampling technique may influence virus count. To identify false negative cases, we combined serological diagnoses with RIDT.^24^ Given that false negative RIDT results lead to misclassification of influenza-related outcomes, the effectiveness of vaccination may be underestimated.

Although serological diagnosis can be used to confirm influenza in cases with negative RIDT results, the definition of influenza infection should be considered. The adjusted OR was lowest when a four-fold increase in HI titers was used for serological diagnosis; however, this OR for four-fold increase in HI titers is considered to be an overestimation of vaccine effectiveness, because subjects with a seroprotective HI titer are unlikely to have a four-fold increase in titer after infection compared to those without a seroprotective HI titer. We therefore concluded that a two-fold increase in HI titer was a better index than a four-fold increase for confirming subclinical influenza infection in the present study. RIDT-diagnosed influenza and RIDT-negative ILI with serologically diagnosed influenza 2, which was considered a false negative result for the RIDT test, was defined as probable influenza, which was the main outcome.

Because protective HI titers represent the level at which approximately 50% of subjects will be protected,^25^ a value of 69% for antibody efficacy against probable influenza might be considered beneficial. Antibody efficacy is strongly influenced by the degree of similarity between the vaccine strains and the epidemic virus. In the 2009/10 pandemic season, when vaccine and virus strains were perfectly matched—as the vaccine had been made using the circulating viral strain—antibody efficacy among pregnant women was reported as 91%.^11^ In the 1991/92 season, when the virus strains were antigenically similar to vaccine strains, the antibody efficacy for A/H3N2 was 86% among healthy adults.^9^ Another study reported that the antibody efficacy for A/H3N2 among institutionalized elderly individuals was 65% in the 2002/03 season, when only 42% of the A/H3N2 isolates were antigenically identical to the vaccine strain.^10^ In the present study season, vaccine and circulating viral strains were well matched antigenically; therefore, antibody efficacy was expected to be higher than our findings suggest. This discrepancy may be due to the fact that influenza infection spread more easily in this population than among subjects in studies for the 2009/10 and 1991/92 seasons, as our study subjects resided in a long-term care facility. Additionally, their lower cellular and humoral immunity may have contributed to influenza infection.

The vaccine efficacy against A/H1N1pdm in the present study was estimated to be 18%, which is lower than efficacy reported in another Japanese study (47.6%)^26^ and a European Union study (55%),^27^ both of which were conducted using a test-negative case-control design among healthy adults in the 2010/11 season. Low immunogenicity caused low vaccine efficacy among subjects with SMID in the present study. If the achievement proportion exceeds 70%, then the vaccine efficacy is expected to exceed 48%. Improving the vaccine efficacy in SMID patients will required further studies to clarify the mechanism behind the population’s low immunogenicity.

The main strength of the present study is the prospective follow-up design. We observed body temperature, respiratory symptoms, and general symptoms in each subject throughout the influenza season. This active follow-up allowed for virological confirmation of the circulating virus strain in the institution. Further, to enhance the outcome accuracy, we used a combination of RIDT and serological diagnosis, which enabled accurate estimation of antibody efficacy. However, a major limitation to the present study warrants mention. Our sample size was too small to detect statistical significance of antibody efficacy, although the analysis had a statistical power of at least 80% to detect a seroconversion proportion >40%. Our study was also single-center, so generalizability may be limited because influenza epidemics differ substantially by location, season, and population. Further, there were few other institutions in the area, limiting our population size.

In conclusion, immunogenicity against A/H1N1pdm in subjects with SMID did not improve in the second influenza season after immunization. Antibody efficacy was moderate for probable influenza among SMID subjects, but vaccine efficacy was insufficient due to the reduced immunogenicity of SMID subjects.

## ONLINE ONLY MATERIAL

eTable 1. Crude ORs for each outcome with respect to subject characteristics.
