# Supplementary material for: Immunogenicity and Efficacy of A/H1N1pdm Vaccine Among Subjects With Severe Motor and Intellectual Disability in the 2010/11 Influenza Season
Source: J Epidemiol. 2016 Jun 5;26(6):300–6. doi: 10.2188/jea.JE20150036 (PMC4884898; doi:10.2188/jea.JE20150036)
Supplement: eTable 1. [file je-26-300-s001.pdf]

eTable 1. Crude ORs for each outcome with respect to subject characteristics

		ARI (fever ≥37.8 °C) (n= 43)			ILI (ARI within endemic period) (n=23)			RIDT-diagnosed influenza (n=11)			Serologically diagnosed influenza 1a (n=14)			Serologically diagnosed influenza 2b (n=14)			Probable influenza c (n=15)		
	n	*n*	OR	95% CI	*n*	OR	95% CI	*n*	OR	95% CI	*n*	OR	95% CI	*n*	OR	95% CI	*n*	OR	95% CI
Asthma																			
Without	93	35	1.00	ref.	17	1.00	ref.	8	1.00	ref.	10	1.00	ref.	14	1.00	ref.	11	1.00	ref.
With	10	8	6.63	(1.33-33.01)	6	6.70	(1.70-26.39)	3	4.56	(0.98-21.13)	4	5.53	(1.33-23.01)	4	3.76	(0.94-15.06)	4	4.97	(1.21-20.42)

ARI, acute respiratory illness; CI, confidence interval; HI, hemagglutination inhibition; ILI, influenza-like illness; OR, odds ratio; RIDT, rapid influenza diagnosis test; S1, post-vaccination; S2, end of the season.

[a] HI titer at S2 / HI titer at S1 ≥4

[b] HI titer at S2 / HI titer at S1 ≥2

[c] RIDT-diagnosed influenza and and an RIDT-negative result with serologically diagnosed
